# The physical activity patterns among pregnant women at a tertiary care hospital in, Pakistan

**DOI:** 10.12669/pjms.38.4.4809

**Published:** 2022

**Authors:** Shabnam Nadeem, Aisha Khatoon, Shaista Rasheed, Tazeen Fatima Munim

**Affiliations:** 1 Dr. Shabnam Nadeem Associate Professor Gynae Unit III, Karachi Medical & Dental College, Abbasi Shaheed Hospital, Karachi, Pakistan; 2 Prof. Dr. Aisha Khatoon Gynae Unit III, Karachi Medical & Dental College, Abbasi Shaheed Hospital, Karachi, Pakistan; 3 Dr. Shaista Rasheed Associate Professor Gynae Unit IIII, Karachi Medical & Dental College, Abbasi Shaheed Hospital, Karachi, Pakistan; 4 Prof. Dr. Tazeen Fatima Munim Head of the Department of Obstetrics/Gynaecology, Karachi Medical & Dental College, Abbasi Shaheed Hospital, Karachi, Pakistan

**Keywords:** Physical activity patterns, Pregnancy, Tertiary care Hospital

## Abstract

**Objectives:**

To determine the physical activity patterns among pregnant women at a tertiary care hospital in, Pakistan.

**Methods:**

A cross sectional study was conducted through questionnaire for a span of eight months (from 17^th^ July, 2020 till 20^th^ March, 2021) amongst pregnant women visiting Abbasi Shaheed Hospital for ante-natal visit including paramedical staff having 24 to 28 weeks of gestation. With the written consent of participants, the physical activity was assessed by a validated self-reported Pregnancy Physical Activity Questionnaire (PPAQ) having 32 questions to determine the duration; frequency, intensity and type of physical activity during pregnancy. Participants were asked to select the physical activity and time spent on it per day during the current gestational period. From the PPAQ, average weekly energy expenditure in Metabolic Equivalent of Task (MET-h-week) was calculated. Demographic data including age, ethnicity, socioeconomic status, parity, literacy and working status were recorded.

**Results:**

A Total of 229 participants of different ethnicities were enrolled. House hold and care giving type of activities of moderate to light intensity were commonly practiced by our pregnant women. Whereas multi gravida were involved in light physical activities. The Punjabi and Baluchi women mostly took occupational type of physical activities. Working women were physically more active than household women. Around 186 (80%) of the participants had no idea that they should take antenatal exercises during pregnancy.

**Conclusion:**

The study concluded that house hold and care giving type activities with moderate to light intensity were commonly carried by pregnant women. Majority of them had no knowledge of antenatal exercise.

## INTRODUCTION

In this era, people tend to lead a sedentary lifestyle which is a root cause of many significant chronic diseases. According to WHO new Global Action Plan, the physical inactivity will be reduced by 15% worldwide by the year 2030.^1^ Conversely, the on-going incremental physical inactivity indicates that this goal now seems challenging.

Physical activity is not only beneficial for everyone, regardless of age, sex, race and ethnicity but it also has numerous health benefits for women during pregnancy. Regular physical activity during pregnancy helps control excessive weight gain and reduce risk of developing Gestational Diabetes, Pre-eclampsia (PE), Hyperlipidemia, and Preterm birth. It also has positive effects on the psychological health of pregnant women.^2^

According to American College of Sports Medicine, during pregnancy physical activity should be performed for at least 30 minutes at moderate intensity, preferably five times a week or for a total of 150 minutes/week.^3^ Unfortunately, physical activity during pregnancy is quite low all over the world. A recent survey in Germany has reported that 41% women had reduced physical activity during pregnancy.^4^ Another study in Germany reported that only 5.3% women to be physically active during pregnancy.^5^

The data of physical activity during pregnancy in Pakistan is deficient. A study was conducted in Agha Khan university found that 86% of women were sedentary and just 3% performed up to 30 minutes per day in sports and exercise activities during pregnancy.^6^ A study published in JCPSP about relation of health-related practices of expectant mothers during pregnancy and fatigue has shown that 12.8% of them doing exercise on a regular basis.^7^

A cross-sectional study conducted in Lahore has reported that 87% of pregnant women had inadequate knowledge of physical activity during pregnancy.^8^ Reduced levels of physical activity during pregnancy might be due to fear of adverse fetal and maternal outcome. Literature has reported that low energy, shortness of breath, backache, and fear of harm to baby factors, history of abortion, or infertility treatment may contribute to reduced physical activity during pregnancy.^9^ Multiple factors of our society such as culture, joint family system and many prevailing myths that physical activity during pregnancy can harm fetus, are also major obstacles in opting for physical activities.

In most of the public sector hospitals, due to work load the health care providers while taking history do not much focus on physical activities being undertaken by the patients during pregnancy. A very little emphasis is being put on this which ultimately leads to lack of awareness among patients.

The aim of the study was to determine physical activity patterns in pregnancy at a tertiary care hospital. This information is helpful for health care providers to counsel the pregnant women and their immediate family members regarding importance of physical activities during pregnancy.

## METHODS

This was a cross-sectional study, conducted at Gynae /Obst department of Abbasi Shaheed Hospital from 17^h^ of July 2020 till 20^th^ March 2021. Ethical approval was obtained from Ethical and Scientific Review Committee of KM&DC (ESRC/KM&DC/048/19, 17-07-2020). The participants were recruited from Abbasi Shaheed Hospital ante-natal clinic including paramedical staff. Patients having 24 to 28 weeks of gestation were recruited through non-probability convenient sampling technique. Subjects, who were febrile, had associated medical disorders, obstetrical complication, multiple gestation or more than forty years of age were excluded from study.

The sample size of n=229 was calculated using the WHO sample size calculator. The reference study used was published in Polish Annals of Medicine.^10^ The calculation performed by considering 18% of physical activity with 95% confidence level and 5% specific precision level.

After taking written informed consent from participants, data were collected through proforma contains demographic information like age, parity, education status, ethnicity, working status and socioeconomic status.

The physical activity was assessed by Pregnancy Physical Activity Questionnaire (PPAQ).^11^ It is a validated self-reported questionnaire having 32 questions to determine the duration; frequency, intensity and type of physical activity practiced during pregnancy. It provides a comprehensive assessment of four domains of physical activity including’’ Sports and Exercises’’ (n=8), House hold and Care giving’’ (n=16), Transportation’’ (n=3) and’’ Occupation’’ (n=5). Participants asked to select the physical activity and time spent on it per day during the current gestational period. The duration ranged from 0 to 6 maximum hours a day. From the PPAQ, average weekly energy expenditure in Metabolic Equivalent of Task (MET-h-week) was calculated. 11

Data was analyzed by SPSS version 23. Continuous variables like age, were presented as Mean, Standard Deviation, Median, Range, 95%CI whereas categorical variables like ethnicity, physical activity patterns were presented in frequency and percentages. A Chi-square test was applied to evaluate the association of physical activity variables with demographics. A p= value<0.05 was considered as statistically significant.

### Importance of study:

To determine physical activity patterns in pregnancy enabling health care providers to counsel pregnant women and their immediate family members about the importance of physical activities during pregnancy. This would help them in controlling excessive weight gain and reduce risk of Gestational Diabetes, Pre-eclampsia (PE), Hyperlipidemia, and Preterm birth.

## RESULTS

A total of 229 participants were enrolled. The mean age was 27.4±4.88SD. Most subjects were highly educated, 120 (52.14%) and 28 (12.2%) were illiterate. Most of our participants were mother of more than one child122 (53.3%). Majority belonged to Urdu speaking ethnicity177 (77.3%) and 192 (83.8%) were household women. 119(52%) of participants belonged to middle class family.

**Fig.1:**
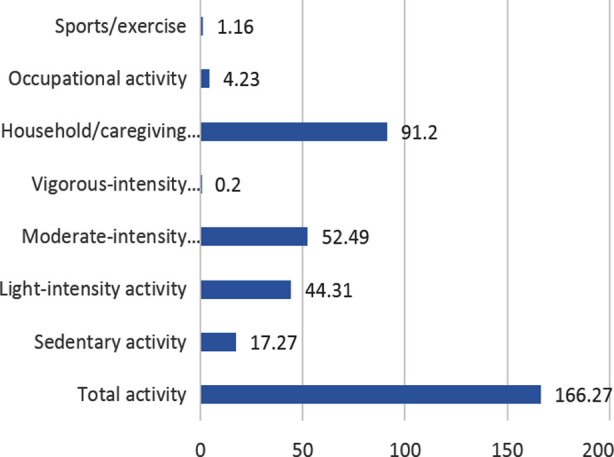
Average Time spent by each physical activity among participants.

As per analysis of PPAQ, household and care giving activities were commonly carried by our study population. Regarding intensity, moderate to light intensity physical activities were mostly practiced during pregnancy (Fig.1).

Among 186 (80%) of the participants had no idea that they should take antenatal exercises during pregnancy. Only 44 (19 %) of participants did exercise in pregnancy and they chose walking for 30 minutes as a preferred form of activity.

Multigravida were more engaged in light intensity activity as compared to primigravida (p value=0.003). When all activities summed up, multigravidas were more physically active as compare to primigravida and grand multi gravida but there was no significant difference among them. (P-value > 0.05) [Table-I].

**Table-I:** Type and intensity of physical activity categorized as per Parity.

	Primi (n=86)	Multi (n=122)	Grand multi (n=20)	P-value
Total activity	110.04 ± 94.91	123.57 ± 101.15	103.37 ± 42.03	0.49
***By intensity***				
Sedentary activity	16.25 ± 22.87	13.64 ± 13.83	23.34 ± 19.95	0.59
Light intensity activity	35.22 ± 30.72	49.33 ± 32.45	53.96 ± 26.81	0.003*
Moderate intensity activity	57.00 ± 44.57	52.40 ± 47.47	36.16 ± 20.60	0.17
Vigorous intensity activity	-	0.38 ± 2.47	-	0.28
***By type***				
Household caregiving activity	86.97 ± 64.81	96.14 ± 67.69	82.99 ± 34.38	0.49
Occupational activity	3.41 ± 8.31	4.60 ± 9.97	5.51 ± 14.74	0.58
Sports exercise activity	1.16 ± 2.49	1.31 ± 3.52	0.32 ± 0.62	0.4

* P-value < 0.05 will be considered as significant.

Punjabi women were more involved in occupational activities. (P value=0.007). The Baloch females were more involved in physical activities as compared to other groups (p-value >0.05) [Table-II]. Working women were physically more active than household women (p value =0.001) [Table-III].

**Table-II:** Type and intensity of physical activity categorized as per ethnicity.

	Urdu speaking (n=177)	Sindhi (n=5)	Punjabi (n=21)	Pathan (n=23)	Balochi (n=3)	P-value
Total activity	119.69 ± 100.75	62.91 ± 27.36	112.78 ± 93.85	96.78 ± 46.05	177.79 ± 71.78	0.4
***By intensity***						
Sedentary activity	17.81 ± 25.35	5.01 ± 5.38	17.95 ± 24.05	12.53 ± 13.78	177.79 ± 71.78	0.36
Light intensity activity	45.19 ± 33.19	22.20 ± 9.02	43.43 ± 34.63	40.66 ± 21.96	63.58 ± 23.42	0.42
Moderate intensity activity	54.28 ± 47.96	33.20 ± 19.31	47.44 ± 40.32	44.35 ± 24.51	76.31 ± 23.87	0.55
Vigorous intensity activity	0.26 ± 2.05					0.93
***By type***						
Household caregiving activity	94.05 ± 68.14	52.04 ± 21.72	80.47 ± 62.17	82.06 ± 34.95	133.29 ± 40.06	0.34
Occupational activity	3.78 ± 9.01	5.46 ± 8.47	11.40 ± 16.98	1.13 ± 4.06	2.68 ± 4.64	0.007*
Sports exercise activity	1.33 ± 3.19	-	0.92 ± 3.01	0.43 ± 1.57	0.53 ± 0.46	0.57

* P-value < 0.05 will be considered as significant.

**Table-III:** Type and intensity of physical activity categorized as per working status.

	House hold (n=192)	Part time job(n=37)	P-value
Total activity	116.01 ± 97.39	117.65 ± 82.95	0.92
By intensity			
Sedentary activity	17.32 ± 24.77	17.03 ± 21.06	0.94
Light intensity activity	45.35 ± 32.49	38.89 ± 29.32	0.26
Moderate intensity activity	53.43 ± 46.37	47.60 ± 36.36	0.47
Vigorous intensity activity	0.24 ± 1.98	-	0.45
By type			
Household caregiving activity	93.86 ± 65.42	77.36 ± 57.56	0.15
Occupational activity	1.21 ± 4.67	19.92 ± 14.06	0.001*
Sports exercise activity	1.07 ± 3.00	1.64 ± 2.97	0.29

* P-value < 0.05 will be considered as significant.

Participants belong to middle to upper class socioeconomically status were more physically active than lower socioeconomically group, p value=0.001 and same group were more engaged in household and care giving activities, p value=0.001 [Table-IV].

**Table-IV:** Type and intensity of physical activity categorized as per income status.

	Lower class <25k (n=107)	Lower middle class>25k---65k (n=72)	Upper middle class >65k—250k (n=47)	High class.>250k (n=2)	P-value
Total activity	97.58 ± 76.14	111.98 ± 86.18	157.16 ± 123.03	289.68 ± 164.76	0.001*
***By intensity***					
Sedentary activity	12.11 ± 20.42	17.64 ± 22.96	26.30 ± 28.74	63.09 ± 41.45	0.001*
Light intensity activity	41.41 ± 29.32	43.52 ± 28.40	50.97 ± 41.00	68.80 ± 47.41	0.25
Moderate intensity activity**	43.77 ± 34.00	48.37 ± 40.17	74.89 ± 60.49	129.24 ± 71.40	0.001*
Vigorous intensity activity	-	0.50 ± 2.97	0.22 ± 1.52	-	0.34
***By type***					
Household care giving activity	79.36 ± 52.43	88.12 ± 57.04	118.88 ± 86.06	171.68 ± 97.26	0.001*
Occupational activity	3.80 ± 9.18	3.05 ± 8.15	5.77 ± 11.75	33.43 ± 7.67	0.001*
Sports exercise activity	0.75 ± 1.44	0.93 ± 3.39	2.04 ± 4.02	10.58 ± 4.37	0.001*

* P-value < 0.05 will be considered as significant

## DISCUSSION

This study was focused on the pregnant women visiting to a tertiary care hospital to assess the level of physical activity during pregnancy. The results showed that the total physical activity score obtained on PPAQ was 166.27/week (approx. 23.75METh/d). These findings were quite similar with studies conducted in Tigray (20.2 METh/d^12^, USA (25.4 METh/d)^13^ but less as compared to France (29 METh/d).^14^ This might be due to different methods used to assess physical activity during pregnancy among these countries.

In our study, women spend more energy on household and care giving activities (91 METh/week). In contrast with other studies conducted in Nigeria^15^ and Tigray^12^ which were 63.4 METh/week and 69.4 METh/week. This difference is due to different cultures like joint family system in which our women besides taking care of their family have also to look after their elders.

Further analysis of parity on PPAQ, it was observed that pregnant women having more than one child were more physically active than the women experiencing it for the first time. These findings are consistent with studies conducted in Tigray^12^, Iowa state (USA), 13Nigeria,^15^ Brazile.^16^ This is because multiparous women have to look-after their children. On the other hand, the reason for physical inactivity in primigravida may be due to their parents and elder’s advice to avoid physical activity during pregnancy as it can cause backache and labor pains as well as there are many mythical believes prevailing in our society which says to avoid outdoor activities in first pregnancy.

Education level of women has a strong impact on their involvement in physical activity during pregnancy. It was observed that fifty two percent of educated women were more physically active as compared to uneducated women. These findings are similar with other studies conducted in Tigray^12^, Australia^17^ and Rio Grande.^18^ This might be due to the fact that highly educated women have more access to information through internet and social media.

It was observed that physical activity score belonging to upper middle to high socio-economic status were higher than women belonging to lower socio-economic group (p value=0.001). The possible reasons are that sample size of each study group was not equal and hence need further studies to make difference. These findings are not consistent with other studies showing mixed results.^19^

Eighty percent of our study participants were completely unaware of the benefits of physical exercise in pregnancy. Only nineteen percent participants of our study knew the importance of physical activities and walked for 30 minutes for physical exercise. Our findings are similar with study conducted in Lahore, Pakistan in which eighty seven percent of pregnant women had inadequate knowledge and negative attitude towards ante natal exercise.^8^ Our findings are not consistent with the study conducted in Africa reported that eighty seven percent of pregnant women were aware of the benefits of physical activity during pregnancy. This difference might be due to lack of education, social and cultural differences of our society.^20^

Sixteen percent of our study participants were working women and they preferred to choose sedentary activities at work. This could be due to decreased energy levels because of poor intake during working hours, fear of initiation of any mishap which may cause harm to pregnancy like abdominal pain and leaking. These findings are consistent with other studies conducted in Hamadan (Iran).^21^

Urbanization plays a pivotal role on physical activity of pregnant women. It was found that women coming from Punjab and Baluchistan were more involved in occupational activities, but the impact of this finding was insignificant as the main population involved in study was Urdu-speaking. Our findings are inconsistent with the research conducted in Poland, reported that women in urban areas are more engaged in occupational activities as compared to women coming from rural area.^22^

### Limitations:

**D**ue to lesser understanding, few of our participants had difficulty on time management of physical activities.

## CONCLUSION

The study concluded that house hold and care giving type activities with moderate to light intensity were commonly carried by pregnant women. Majority of them had no knowledge of antenatal exercise.

### Recommendations:

Health care providers should develop a health care programme in which physical activity must be an integral component. It is their responsibility to encourage pregnant women to take ante natal exercise classes, counseling on physical activity, and disseminate information on the exercises suitable for pregnant women.A structured program including Guidelines regarding physical exercises during pregnancy should be developed at national level.

### Author`s Contribution:

**SN:** Conception, design, and prepared the manuscript. She is also responsible for integrity of submitted research work.

**SR:** Data collection Interpretation of data.

**AK:** Data analysis and preparation of the write up.
